# Benign metastasizing fumarate hydratase (FH)-deficient uterine leiomyomas: clinicopathological and molecular study with first documentation of multi-organ metastases

**DOI:** 10.1007/s00428-024-03806-8

**Published:** 2024-04-20

**Authors:** Xiaoxue Yin, Xiaoxia Wei, Ruqaiya Al Shamsi, Fatima S. Ali, Faiza Al Kindi, Xingming Zhang, Jiayu Liang, Xiuyi Pan, Mohammed Al Masqari, Linmao Zheng, Qiao Zhou, Abbas Agaimy, Ni Chen

**Affiliations:** 1 Department of Pathology, West China Hospital, Sichuan University, Chengdu, China; 2 Laboratory of Pathology, West China Hospital, Sichuan University, Chengdu, China; 3 Department of Pathology, West China Second University Hospital, Sichuan University, Chengdu, China; 4 Department of Pathology, The Royal Hospital, Muscat, Sultanate of Oman; 5 Department of Radiology, The Royal Hospital, Muscat, Sultanate of Oman; 6 Department of Urology, West China Hospital, Sichuan University, Chengdu, China; 7 Institute of Pathology, Erlangen University Hospital, Comprehensive Cancer Center, European Metropolitan Area Erlangen-Nuremberg (CCC ER-EMN), Friedrich Alexander University of Erlangen-Nuremberg, Erlangen, Germany

**Keywords:** Fumarate hydratase-deficient leiomyoma, Multi-organ metastases, Lung, Kidney, HLRCC, Hereditary genital cancer

## Abstract

Leiomyoma is the most prevalent benign tumor of the female reproductive system. Benign metastasizing leiomyoma (BML) is a rare phenomenon that presents at distant sites, typically the lungs, exhibiting histopathological features similar to the primary uterine tumor in the absence of malignancy features in both. Fumarate hydratase-deficient uterine leiomyoma (FH-d UL) is an uncommon subtype among uterine smooth muscle tumors (0.5–2%), showing distinctive histomorphology and *FH* inactivation. The majority of FH-d ULs are sporadic, caused by somatic *FH* inactivation, while a minority of cases occur in the context of the hereditary leiomyomatosis and renal cell carcinoma (HLRCC) syndrome caused by germline *FH* inactivation. Metastasizing FH-d UL has not been well documented and might be under-reported. Here, we present two cases (21- and 34-year-old females) who presented with metastasizing FH-d UL after myomectomy/hysterectomy with histologically proven multiple lung metastases in both, in addition to multi-organ involvement in one case (cervical-thoracic lymph nodes, left kidney, perihepatic region, left zygomatic bone, and soft tissues). Pathological examination confirmed FH-d leiomyomas in the primary/recurrent uterine tumors, multiple lung lesions, and a renal mass. The minimal criteria for diagnosis of leiomyosarcoma were not fulfilled. Genetic testing revealed germline pathogenic *FH* variants in both cases (c.1256C > T; p.Ser419Leu in Case 1 and c.425A > G; p.Gln142Arg in Case 2). These novel cases highlight a rare but possibly under-recognized presentation of FH-d BML. Our study suggests that FH-d BML cases might be enriched for the HLRCC syndrome.

**Supplementary Information:**

The online version contains supplementary material available at 10.1007/s00428-024-03806-8.

## Introduction

Uterine leiomyoma is the most common tumor of the female reproductive system, with a prevalence of up to 70% in women [1]. Genetic alterations associated with uterine leiomyoma are heterogeneous and they include mutations of mediator complex subunit 12 (*MED12*), overexpression of high mobility group AT-hook 2 (HMGA2), and inactivation of the fumarate hydratase (*FH*) gene [2]. Fumarate hydratase-deficient uterine leiomyoma (FH-d UL) is uncommon and exhibits unique histopathological and molecular changes, representing 0.5–2% of consecutive uterine smooth muscle tumors [3, 4]. Despite being histologically benign, uterine leiomyoma can rarely metastasize, mostly to the lung, a phenomenon referred to as “benign metastasizing leiomyoma (BML).” BML is manifested as single or multiple well-differentiated smooth muscle nodules involving the lung and rarely multiple sites [5, 6]. Definitionally, BML displays concordant histology between the primary uterine tumor and the metastases and lacks histological evidence of malignancy in both primary and metastatic lesions. However, FH-d BML has not been well documented before and might be under-recognized or under-reported. In this study, we describe two well-characterized FH-d UL cases with metastases to the lung and one case with additional multi-organ involvement, including the kidney.

## Material and methods

The two cases were retrieved from the authors’ files. The tissue specimens were fixed in formalin and processed routinely for histopathology. Due to the consultation nature of the cases, immunohistochemistry (IHC) was performed in different laboratories and the stains applied varied from case to case (details of the staining protocols and antibody sources are provided in the supplementary tables).

## Molecular testing

In Case 1, after genetic counselling, whole-exome sequencing was performed on the collected peripheral blood sample of the patient and her mother. Representative sections of formalin-fixed paraffin-embedded tumor were utilized for DNA extraction, and PCR was employed to validate *FH* alterations, using the primers 5′-CTCTCTCTCTCTCTCACTCACT-3′ (FP) and 5′-TCTCAAACACTGATCCACTTGTCTCTT-3′ (RP). The PCR products were subsequently identified through Sanger sequencing.

In Case 2, molecular testing (targeted next-generation sequencing) was performed after genetic counselling and informed consent on collected peripheral blood sample using the Ion Ampliseq Comprehensive Cancer Panel (409 genes on Ion S5 platform).

## Results

### Case 1

#### Case description

A 21-year-old female with menorrhagia was diagnosed with multiple uterine leiomyomas in 2012, leading to myomectomy. Recurrent tumors prompted myomectomy in 2019 and 2021. In July 2022, the patient developed premenstrual chest and back pain, and CT revealed numerous nodules in both lungs, left kidney, perihepatic region, left zygomatic bone, bilateral neck, bilateral erector spinae muscle, psoas major, thoracoabdominal muscle layers, and intercostal spaces. Laparoscopic partial left nephrectomy and wedge resection of the right middle lobe of the lung were performed.

Exploration of her medical history revealed the patient’s mother had uterine leiomyomas two decades ago. Subsequently, her mother developed metastatic kidney cancer in 2022 and passed away in 2023. Genetic testing on her mother’s peripheral blood sample confirmed the presence of the same *FH* germline mutation (see molecular results below). Moreover, the patient’s younger sister died of papillary renal cell carcinoma of the left kidney in 2014.

#### Imaging and pathological findings

**Fig. 1:**
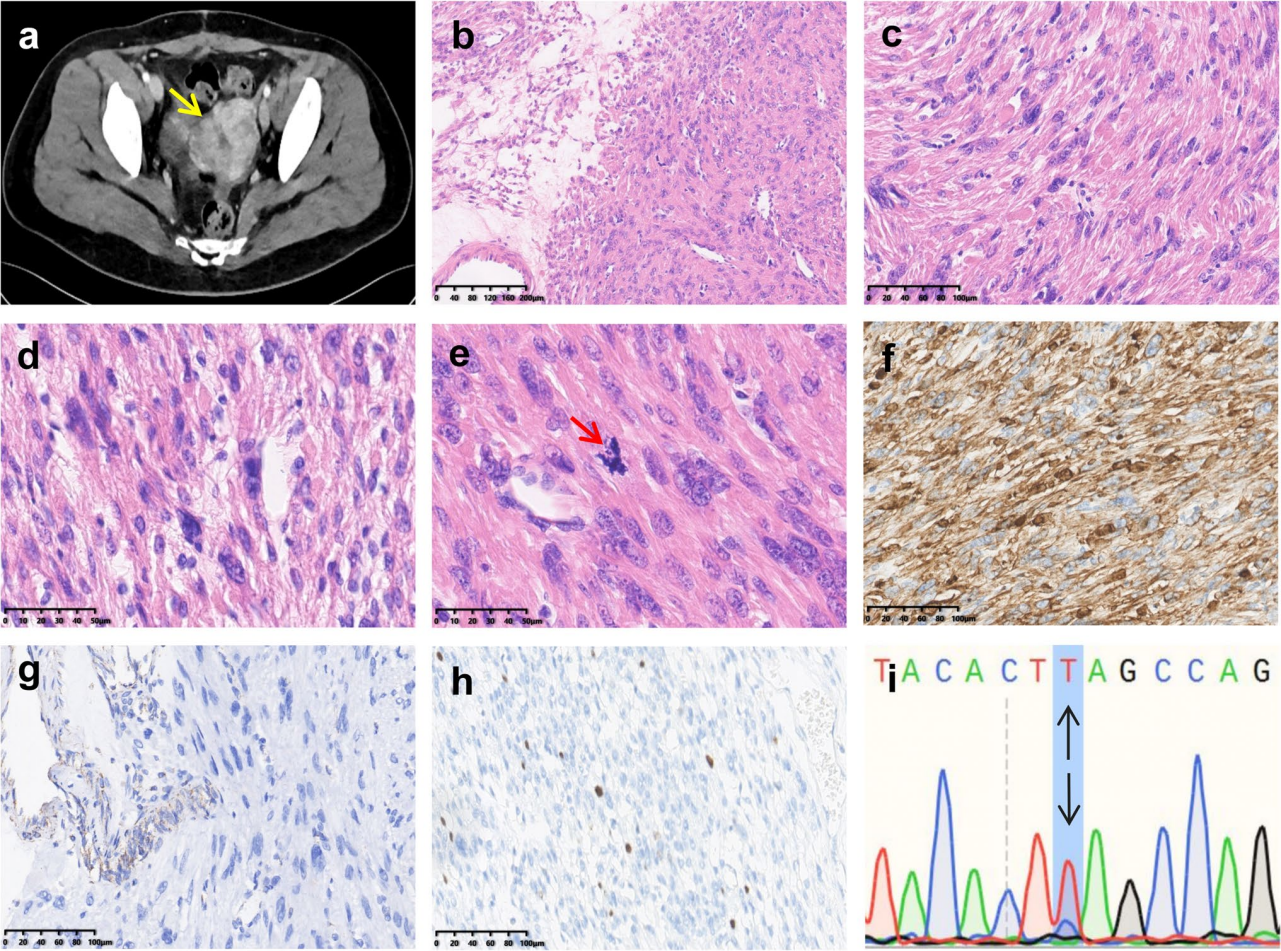
Pathological and molecular genetic features of the recurrent uterine leiomyoma in 2019 of Case 1. **a** CT scan showing irregular enlargement of the uterus, with multiple soft tissue density nodular shadows. **b** The tumor cells were arranged in a woven pattern, with antler blood vessels and interstitial edema. **c** Mild cellular atypia in tumor cells, with abundant eosinophilic cytoplasm, and hyaline cytoplasmic globules. **d** Bizarre nuclei, eosinophilic nucleoli with perinucleolar halos, and prominent eosinophilic/fibrillary cytoplasm. **e** Mild nuclear atypia with rare scattered mitoses. Tumor cells were positive for **f** desmin and negative for **g** FH. **h** The Ki-67 proliferative index was less than 5%. **i** The tumor showed the *FH* mutation

CT imaging revealed irregular enlargement of the uterus with multiple soft tissue density nodular shadows (Fig. 1a). Grossly, the uterine masses exhibited expansile growth within the myometrium, ranging from 0.2 to 10 cm in diameter. Microscopically, the tumors displayed intersecting fascicles of spindle cells with variable edematous stroma in-between and numerous staghorn vessels (Fig. 1b). Tumor cells showed mild atypia with medium or abundant dense eosinophilic cytoplasm, occasionally showing eosinophilic, hyaline cytoplasmic globules. Nuclei were oval or cigar-shaped, with eosinophilic nucleoli and perinuclear halos. Bizarre cells, characterized by large eosinophilic nucleoli, and heterogeneous chromatin were scattered in the background. Mitotic activity did not exceed 2/10 high-power fields (HPFs) (Fig. 1c–e). No foci of necrosis were seen. IHC showed strong expression of desmin, loss of FH expression, and a Ki-67 proliferation index of < 5% (Fig. 1f–h).

**Fig. 2:**
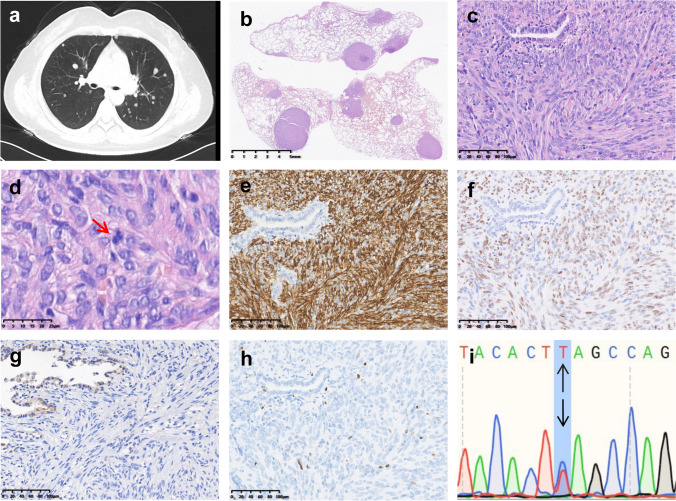
FH-d UL with lung metastasis in Case 1. **a** CT image showing multiple nodules with smooth, clear borders and uniform density in both lungs. **b** Numerous round or oval tumor nodules in the lung excision. **c** Entrapped native alveolar glands were observed within the tumor. **d** A mitotic figure is marked by a red arrow. Tumor cells were positive for **e** desmin and **f** ER and negative for **g** FH. **h** The Ki-67 index was less than 5%. **i** The same mutation of *FH* gene in the tumor

On CT scan, the lung lesions presented as multiple rounded nodular shadows with increased density in both lungs, measuring 0.5–0.8 cm in diameter and featuring clear borders (Fig. 2a). Histologically, well-delineated multiple nodules were seen in the right middle lobe wedge resection at the low magnification (Fig. 2b). Tumor cells exhibited similar morphology as the uterine leiomyomas, with scattered entrapped alveolar glands within the tumors. The cells showed moderate density and occasional bizarre nuclei. Mitotic figures were less than 0–1/10 HPFs (Fig. 2c, d). IHC demonstrated desmin, PR and ER expression, loss of FH, and Ki-67 < 5% (Fig. 2e–h).

**Fig. 3:**
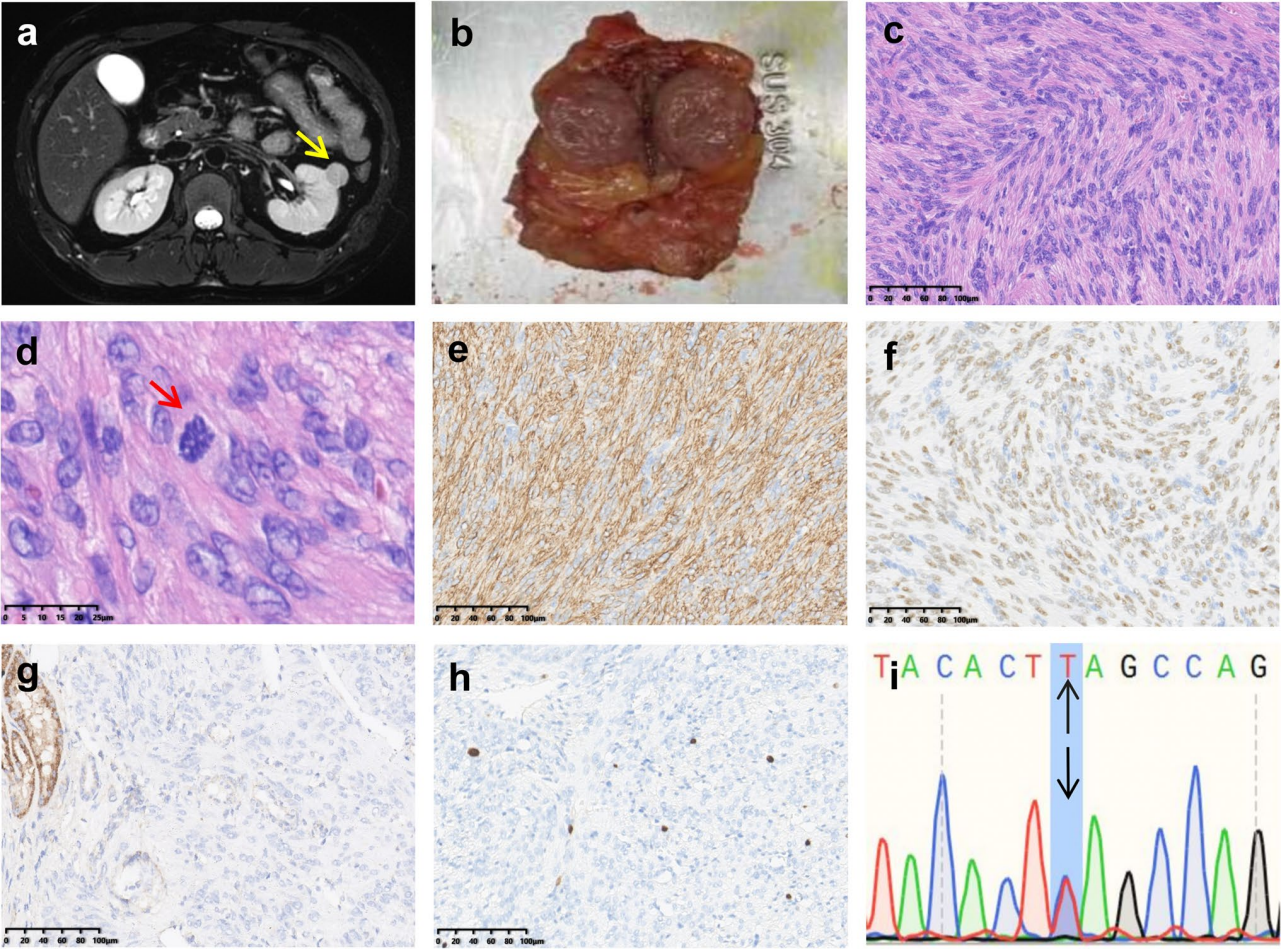
FH-d leiomyoma of the kidney of Case 1. **a** A circular, slightly dense nodule with a clear boundary and moderate enhancement in the upper pole of the left kidney. **b** The tumor was grayish white and tough in texture. **c** Spindle-shaped tumor cells arranged in bundles, exhibiting well-differentiated features with mild cellular atypia. **d** Mild nuclear atypia with rare scattered mitoses. Immunohistochemical staining was performed for **e** SMA, **f** ER, **g** FH, and **h** Ki67. **i** The same* FH* gene mutation was confirmed in the tumor

The renal mass displayed a slightly denser nodular shadow in the mid-portion of the left kidney with clear borders and moderate contrast enhancement on CT scan (Fig. 3a). Grossly, a 2.1 cm × 1.7 cm tumor adjacent to the renal capsule, exhibiting a gray-white appearance and firm texture, was seen (Fig. 3b). Microscopic analysis revealed a well-demarcated tumor with spindle cells, moderate to abundant cell density, mild pleomorphism, and occasional large nuclei. No bizarre cells or significant mitotic activity was observed (Fig. 3c, d). Necrosis was absent. IHC showed SMA, PR and ER positivity, FH loss, and a Ki-67 proliferation index of 5–10% (Fig. 3e–h). Altogether, ER and PR showed comparable moderate to diffuse expression in all tested uterine, pulmonary and renal lesions (representative examples are shown in the supplementary Figs. 1–8).

#### Molecular findings

Whole-exome sequencing on collected peripheral blood sample revealed the *FH* c. 1256C > T (p.Ser419Leu) germline mutation which was then confirmed by Sanger sequencing on DNA extracted from formalin-fixed paraffin-embedded uterine tumor tissue (Fig. 1i). The same mutation was also confirmed in the blood sample from the patient’s mother. These findings are confirmatory of the hereditary leiomyomatosis and renal cell carcinoma (HLRCC) syndrome in the patient and her family. Sequencing of the lung (Fig. 2i) and renal metastases (Fig. 3i) and corresponding adjacent renal tissue confirmed the presence of the same *FH* c.1256C > T germline mutation.

### Case 2

#### Case description

A 34-year-old lady with primary infertility underwent myomectomy and was diagnosed with atypical leiomyomas. Two years later, she developed a recurrence of leiomyomas for which she underwent hysterectomy and was reported as uterine smooth muscle tumors of uncertain malignant potential (STUMP). A chest CT scan done before the hysterectomy showed multiple bilateral lung nodules. She was then lost to follow-up after the hysterectomy, until she presented 7 years later to the hospital with on and off cough and mild hemoptysis. Her previous chest CT scan was reviewed, and a new CT-PET scan was performed which showed an interval increase in the number and size of the lung nodules. Specifically, the nodules have nearly doubled in number, and most have grown to double their original size (up to 1.8 cm in diameter).

A biopsy obtained from one of the lung nodules was consistent with metastatic FH-d leiomyoma with concordant histology as the primary tumors of the uterus. The case was discussed once in the MDT, and a decision was made to offer the patient hormonal therapy.

**Fig. 4:**
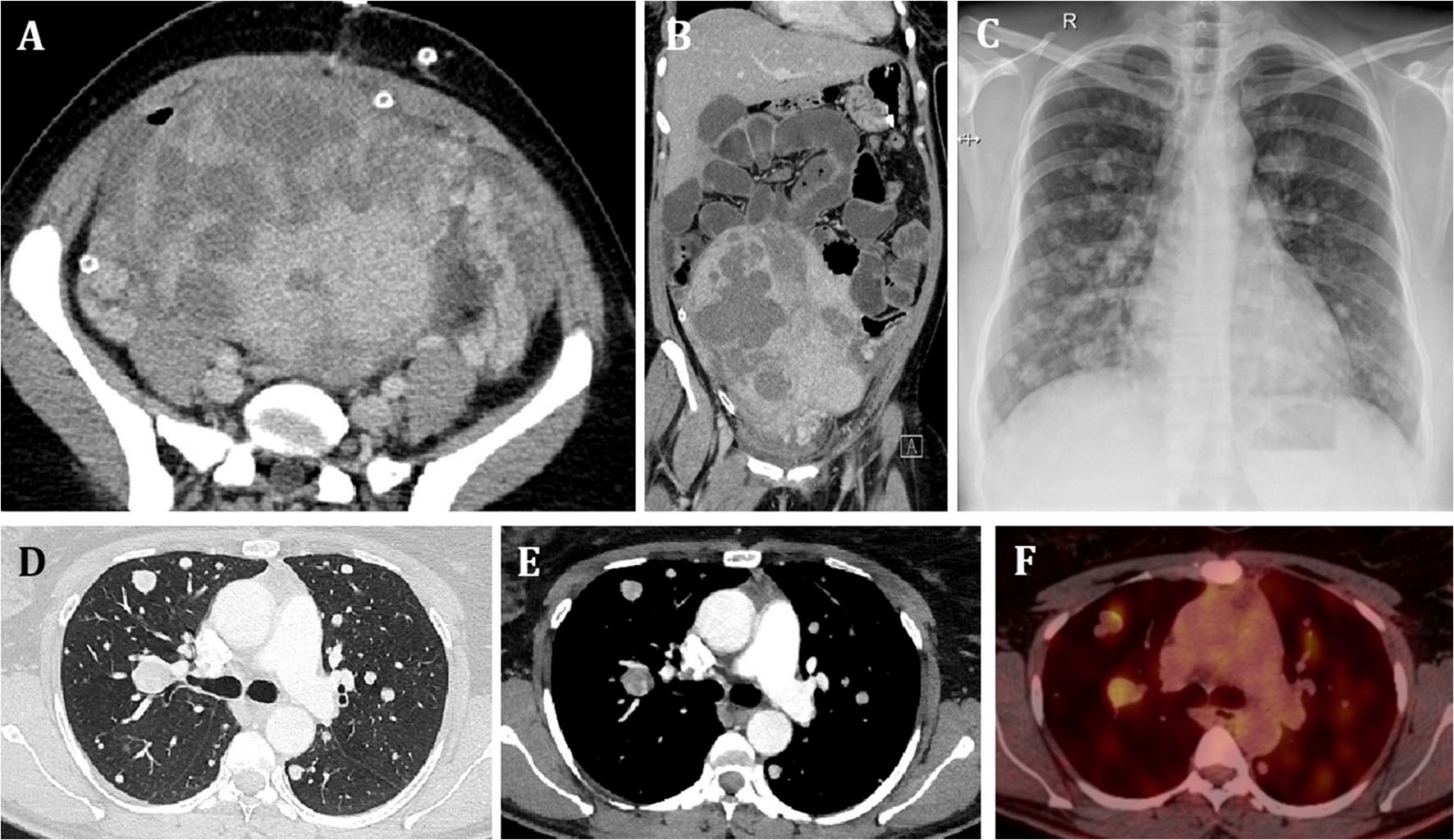
Imaging findings in Case 2. **A**, **B** Axial and coronal images from CT of the abdomen demonstrating the large heterogeneously enhancing leiomyomas. **C** Chest radiograph of the patient showing multiple radio-opaque nodular opacities with a variable size in both lungs. **D** Axial CT of the lung window showing multiple variable-sized randomly distributed lung nodules, typical of metastatic lung nodules. **E** Axial CT lung with a meditational window showing multiple nodules with heterogeneous densities and IV contrast enhancement. **F** Axial CT-PET fused images demonstrating high FDG uptake in the lung nodules

Imaging of the pelvis and abdomen revealed large heterogeneously enhancing uterine masses/nodules (Fig. 4a, b). The thorax x-ray (Fig. 4c) and CT scan (Fig. 4d, e) imaging showed multiple bilateral lung nodules with similar characteristic as described for Case 1 above; some of them were also PET-positive (Fig. 4f).

#### Pathological findings

**Fig. 5:**
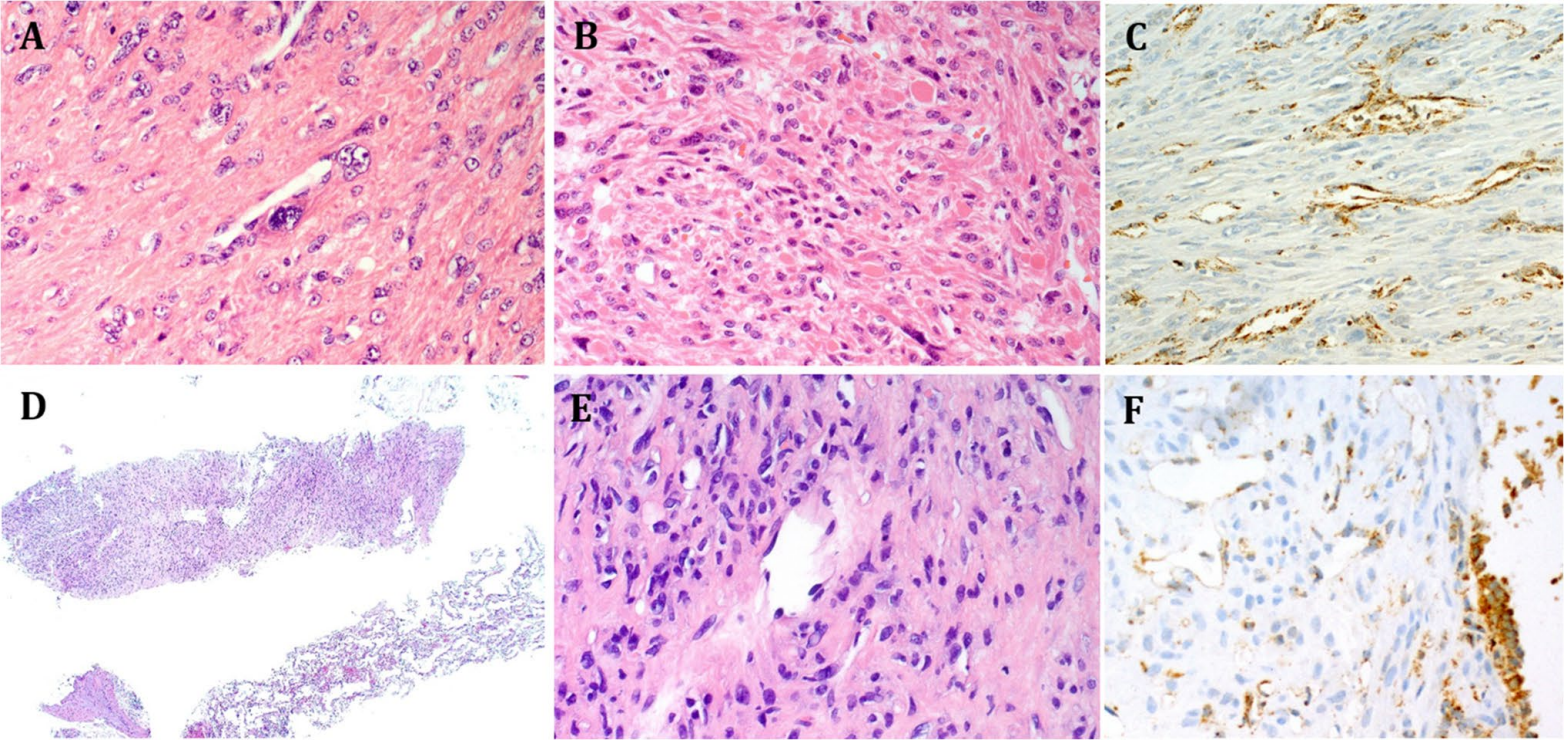
Histomorphological features of uterine and lung tumors in Case 2. The uterine smooth muscle neoplasms of Case 2 showed features characteristic of FH-d leiomyomas with variably enlarged lobulated vesicular nuclei with perinucleolar chromatin clearing/halos (**A**) and interspersed degenerating globular eosinophilic smooth muscle fibers resembling skeletal muscle fibers (**B**). FH IHC confirmed complete loss restricted to the neoplastic cells (**C**). The lung core needle biopsies (**D**) showed mild atypia with nuclear vacuoles but no mitotic activity or necrosis (**E**). FH IHC confirmed complete loss restricted to the neoplastic cells (**F**), note retained granular cytoplasmic reactivity in endothelial cells in the background and in a small bronchiole on the right

Grossly and histologically, the tumors displayed overall similar features as described in Case 1 with moderate cellularity, short-elongated, plump nuclei, and remarkable cytoplasmic eosinophilia with fibrillary quality, focally resembling neuropil-like matrix surrounded by nuclear palisades reminiscent of neuroblastic/schwannian neoplasms. Scattered enlarged or bizarre nuclei with pseudoinclusions, variable perinucleolar halos (Fig. 5a), hyaline globular bodies indicating degenerating muscle fibers, occasionally mimicking skeletal muscle fibers (Fig. 5b), and prominent hemangiopericytoma-like vasculature were the main features observed. The uterine and the pulmonary nodules (Fig. 5d, e) revealed concordant histological pattern/features, and none fulfilled the minimum set of the uterine criteria to be classified as STUMP or leiomyosarcoma. Notably, no diffuse atypia or tumor necrosis were seen, and mitoses were virtually absent (< 2/10 HPFs). All tumors strongly expressed smooth muscle actin and desmin. FH loss was confirmed in the primary and metastatic nodules by IHC (Fig. 5c, f). The expression of the hormone receptors was concordant between the uterine nodules and the lung metastases. Notably, the PR was strongly and diffusely positive in the uterine nodules and in the lung metastases, while the ER was negative in the uterine nodules and only focally and weakly positive in the lung metastases.

#### Molecular findings

Genetic testing of a peripheral blood sample after informed consent revealed a pathogenic heterozygous *FH* variant c.425A > G; p.Gln142Arg. This mutation has been previously reported as likely pathogenic in individuals with multiple cutaneous and uterine leiomyomatosis with or without renal cell cancer and is hence known to be associated with the HLRCC syndrome [7–9].

## Discussion

Leiomyomas can occur in any body site where they originate either from smooth muscle of visceral organs or from vascular wall musculature, including the uterus, digestive tract, skin, and soft tissues. Multiple-organ, multifocal leiomyomas are rare and may represent either independent primaries (sharing a common etiology such as EBV infection in the immunocompromised patients or an inherited causal gene mutation such as *FH* inactivation in the HLRCC syndrome context), or they are considered metastases originating from benign uterine leiomyoma [10]. Benign metastasizing leiomyoma (BML) is an extremely rare disease, initially reported by Steiner in 1939 [11], with over 160 documented cases in subsequent literature [12]. It predominantly affects females with uterine leiomyomas, at an age ranging from 35 to 55 years [13]. The lungs are the most commonly affected sites of metastases, commonly presenting as solitary or multiple bilateral masses with occasional cystic appearance [12]. Diagnosis of BML should be critically rendered and only after critical reevaluation of both the primary and the metastatic nodule/s for evidence of malignancy. By definition, both the primary and the metastatic nodule/s of BML share similar histology and lack evidence of malignancy such as diffuse atypia, significant mitotic activity, and necrosis.

BML with FH defects is exceptionally rare with only two reported cases in the literature [14, 15]. In both instances, lung metastasis occurred. In our study, both patients initially sought treatment for uterine leiomyomas with multiple recurrences and later were found to have multiple lung nodules, in addition to renal and other site involvement. Our cases are novel in that they represent the second documentation of histologically proven FH-d BML in the lungs (both cases) and the first documentation of multi-organ non-pulmonary involvement (verified histologically in the kidney in one case).

FH-d ULs comprise up to 2% of all uterine leiomyomas which in the light of very high prevalence of uterine fibroids in the general population, indicates these tumors are encountered relatively frequently in busy centers [3]. The overall frequency of BML among FH-d UL and (conversely) the frequency of FH-d UL among reported BML cases are unknown, but might be under-reported, given that, including our two cases, only four cases have been reported (only three with histological verification) [14, 15].

The differential diagnostic consideration of our cases is limited in the light of proven *FH* gene mutations/inactivation. Metastasis from genuine uterine leiomyosarcoma (LMS) with FH loss is a possibility that is ruled out in both our cases by conventional histological malignancy criteria. Although initially considered controversial, the occurrence of FH-d uterine LMS has been recently documented. Chapel et al. detected 7 FH-d uterine LMS among 348 screened cases (2%) [3]. The age spectrum of FH-d LMS (range, 42–67 years; median, 56) is significantly higher than in our cases (21 and 34 years). Reported FH-d LMS showed global FH loss, with one case displaying small subclonal LMS within a background leiomyoma, both were FH-d. Detailed clinical data on manifestations of the HLRCC syndrome and germline molecular data were not available for the reported FH-d LMS in the above-cited study [3]. Based on these preliminary observations, the question of whether FH-d uterine LMSs originate from preexistent FH-d LM remains currently unanswered.

The possibility that the extra-uterine nodules in our cases might represent genuine independent primaries in our patients, both with germline disease (HLRCC), needs consideration. To date, FH-d ULs have been restricted to the uterus (in sporadic cases) or the uterus + skin in individuals with the HLRCC syndrome. To our knowledge, non-cutaneous extra-uterine FH-d ULs have not been reported in any other organ. Moreover, the widespread pattern in the lungs involving all lung lobes bilaterally is consistent with metastatic rather than primary disease.

The renal involvement in one of our cases represents a novel oncological pitfall, given that the majority of renal masses in HLRCC patients predominately indicate aggressive RCC as part of the syndrome. A previous case report on HLRCC syndrome has documented an incidental FH-proficient renal monotypic angiomyolipoma indicating the need for histological verification of any renal lesion in the context of HLRCC syndrome to avoid misinterpretation as aggressive RCC on imaging alone, which would result into unnecessary radical nephrectomy or systemic overtreatment [16]. We herein report a new novel finding illustrating renal involvement by FH-d BML of uterine origin in the context of the HLRCC syndrome. This solitary FH-d renal leiomyoma was morphologically similar to the uterine leiomyomas, in line with a BML manifestation, same as the lung nodules. Finally, it is challenging to determine whether the lesions in other soft tissue locations in Case 1 lacking pathological confirmation represent metastatic FH-d BML or other unrelated lesions. However, histological verification of the renal lesion as being unusual FH-d BML strongly argues for the notion that these soft tissue and bone lesions very likely represent the same findings as the renal nodule and the multiple lung lesions.

The *fumarate hydratase gene FH* (mapped to 1q43) is an integral key enzyme in the tricarboxylic acid cycle (the Krebs cycle) which is responsible for the conversion of fumarate to malate. *FH* may be affected by both germline and somatic mutations, with the former associated with the HLRCC syndrome and the latter observed in the majority of sporadic FH-d ULs [17]. In this study, we identified the *FH* c.1256C > T mutation in Case 1 and the c.425A > G mutation in Case 2; both were germline variants. These mutations have been documented in the literature as pathogenic and are associated with HLRCC syndrome [7–9, 18]. Although the two patients did not present with other features of the HLRCC syndrome yet, Case 1 had a strong family history of HLRCC syndrome, and genetic testing on her mother’s blood sample revealed the presence of the same *FH* c.1256C > T mutation.

One last issue, raised by our cases, is whether BML cases with FH loss are enriched for germline disease (HLRCC). Indeed, both our cases and the case reported by Gilhooley et al. had a germline *FH* mutation indicating that three of four reported FH-d BML cases were germline disease. The case reported by Ahvenainen et al. did not have a germline *FH* variant, but only normal ovarian tissue was tested for the *FH* mutation and not a blood sample, so the presence of a germline disease is not reliably ruled out. Overall, these observations are suggestive of FH-d ULs as being more frequently associated with the HLRCC syndrome than sporadic FH-d leiomyoma, but this remains to be verified in future studies.

Despite FH-d UL’s large size, the prognosis seems generally favorable, with rare malignant transformation [14]. However, limited data precludes any reliable prognostic conclusion. A study involving 86 FH-d ULs reported 5 deaths occurring 9–30 years after surgery in patients aged 56–97 years. However, death directly related to tumors is inconclusive due to advanced age of many included patients [19]. FH-d UL, common in young females, often requires uterus-preserving surgery based on size and location. No standardized FH-d UL treatment guidelines exist, and options include observation, surgery, and hormone therapy [20]. Patient prognosis is influenced by extent of tumor metastasis. The interval between hysterectomy and the diagnosis of BML varies, with an average duration of 10 years, ranging from 3 to 20 years [21]. Similarly, patients in this study experienced multiple recurrences after myomectomy and developed multi-organ metastases, 10 years later.

Both of our patients have initially received morcellated myomectomy, suggesting a potential role for this type of conservative surgery in the pathogenesis of BML. However, a review of the previous literature did not support this notion, as BML has been reported across all types of myometrial surgery [12]. Moreover, some cases have occurred without prior myometrial surgery [12].

In summary, we have presented two histologically verified benign metastasizing FH-d ULs, one with multi-organ metastases in addition to lung nodules, occurring in two women with verified *FH* germline mutations. Familiarity of lung pathologists with the morphological patterns of FH-d UL is mandatory to initiate FH testing and uncover potential genetic diseases. Moreover, a biopsy of any kidney mass in patients with the HLRCC syndrome is mandatory to rule out BML in this context to facilitate nephron-sparing surgery and avoid unnecessary radical renal surgery for benign lesions.

### Supplementary Information

Below is the link to the electronic supplementary material.

Supplementary file1 (DOCX 4221 KB)

## Data Availability

The raw data are included in the article, and inquiries can be directed to the corresponding author.
