# Supplementary material for: Benign metastasizing fumarate hydratase (FH)-deficient uterine leiomyomas: clinicopathological and molecular study with first documentation of multi-organ metastases
Source: Virchows Arch. 2024 Apr 20;485(2):223–31. doi: 10.1007/s00428-024-03806-8 (PMC11329531; doi:10.1007/s00428-024-03806-8)
Supplement: Supplementary file 1 — Supplementary file1 (DOCX 4221 KB) [file 428_2024_3806_MOESM1_ESM.docx]

Table 1. Antibodies used for immunohistochemistry in Case 1

| Antibody | Clone | Source | Antibody dilution |
| --- | --- | --- | --- |
| desmin | MX046 | ZGSB-BIO | 1:200 |
| ER | SP1 | ROCHE | Ready-to-use |
| FH | J-13 | SANTA CRUZ | 1:600 |
| Ki-67 | MIB-1 | ZGSB-BIO | 1:200 |
| PR | 1E2 | ROCHE | Ready-to-use |
| SMA | UMAB237 | ZGSB-BIO | 1:100 |

Table 2. Antibodies used for immunohistochemistry in Case 2

| Antibody | Clone | Source | Antibody dilution |
| --- | --- | --- | --- |
| desmin | D33 | DAKO | 1:250 |
| ER | EP1 | DAKO | Ready-to-use |
| FH | J-13 | SANTA CRUZ | 1:50 |
| PR  SMA | PgR636  1A4 | DAKO  DAKO | Ready-to-use  1:200 |

**
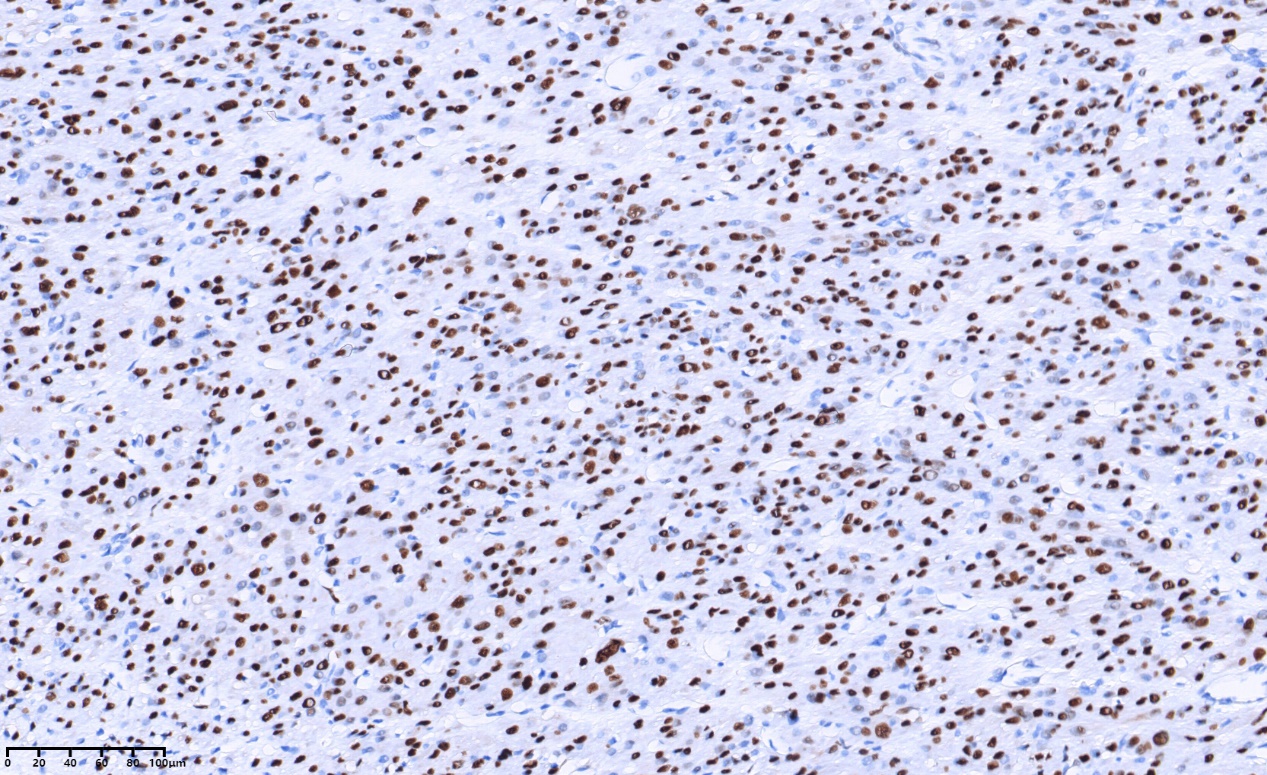
**

Fig.1 ER showed strong diffuse positivity in the uterine lesion.

**
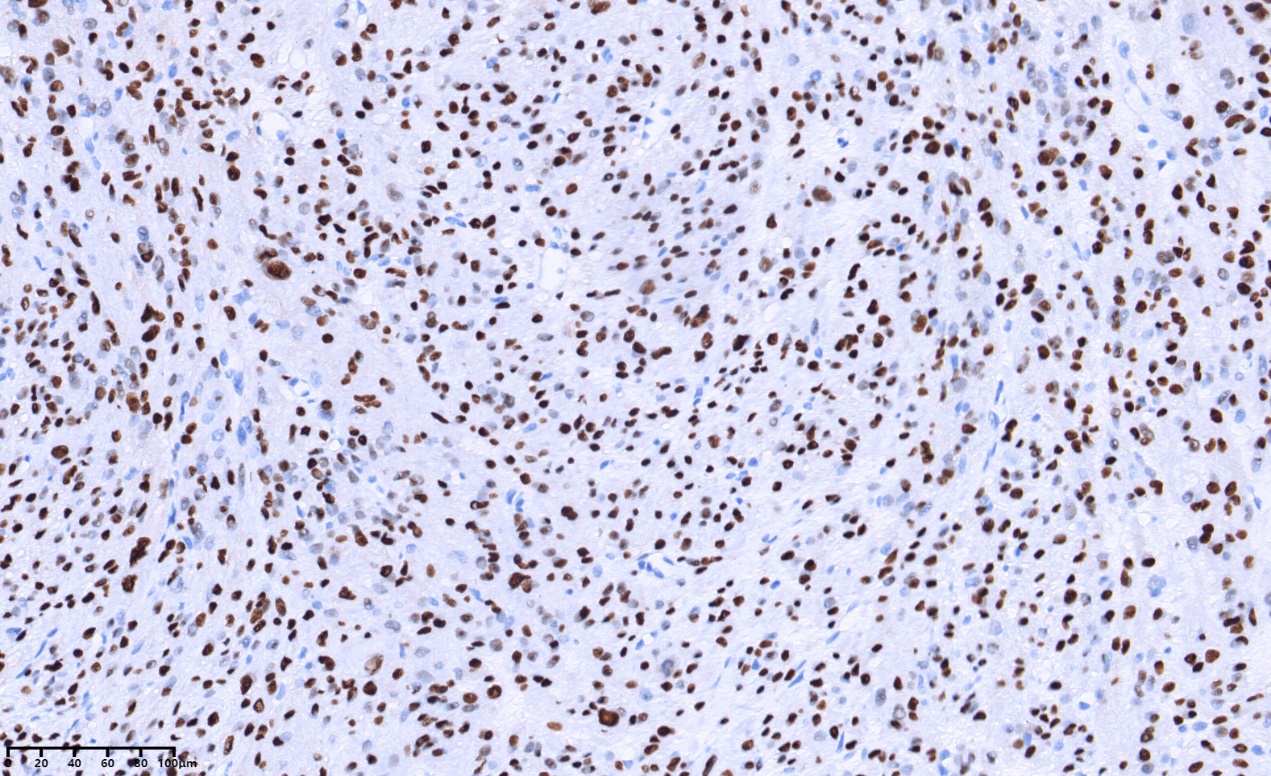
**

Fig.2 PR showed strong diffuse positivity in the uterine lesion.

**
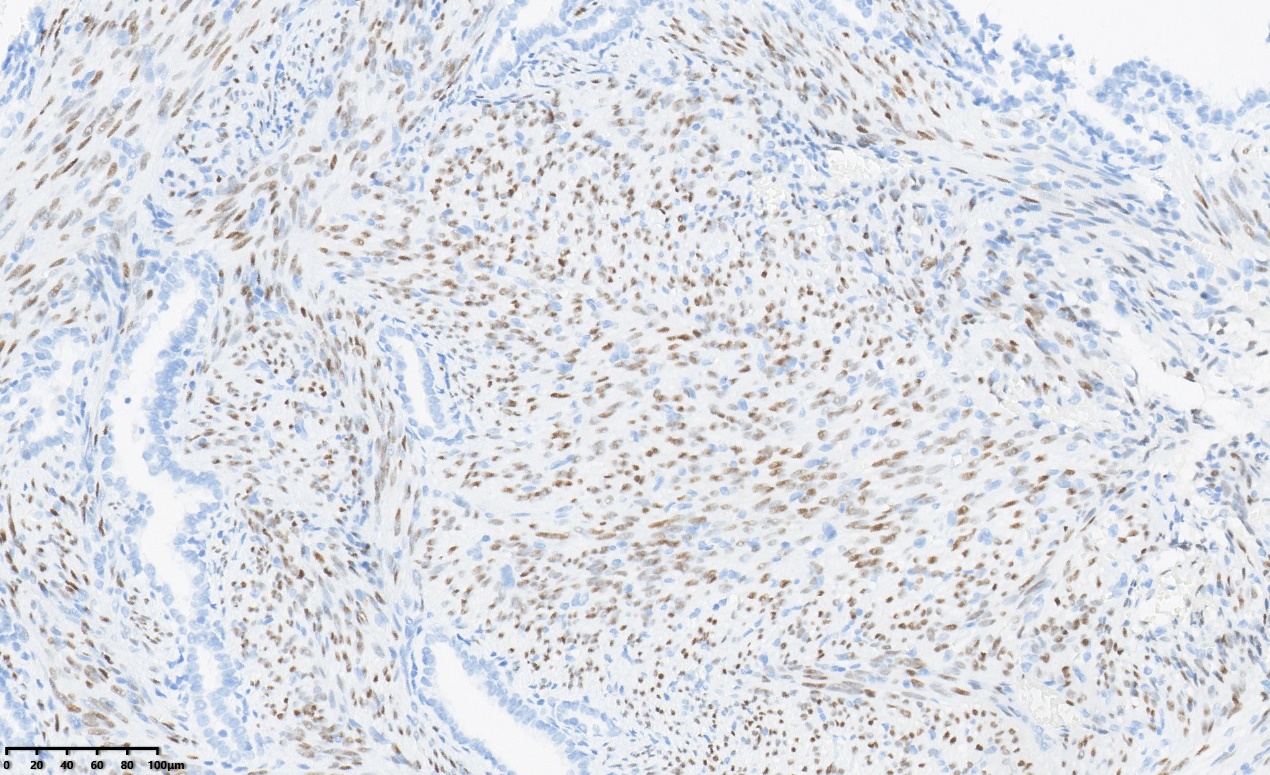
**

Fig.3 ER was moderately positive in the lung metastatic lesion (low power).


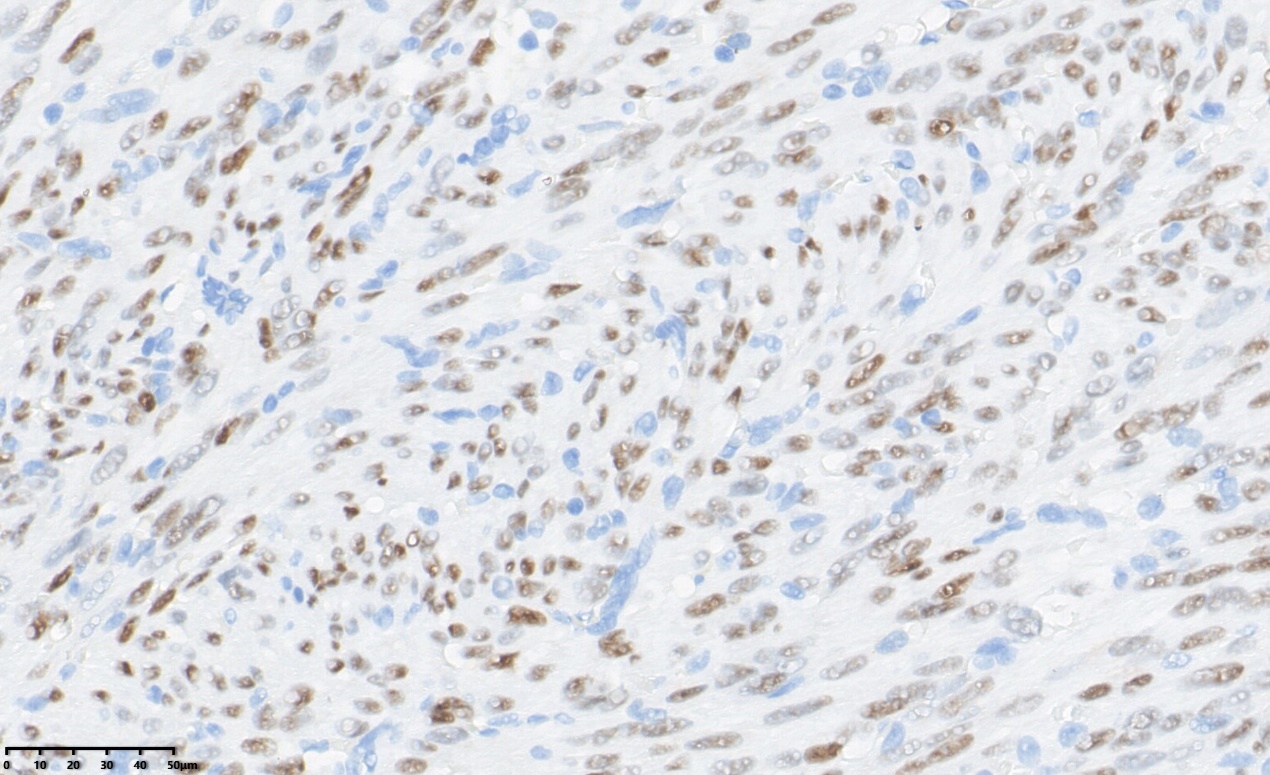


Fig.4 ER was moderately positive in the lung metastatic lesion (high power).


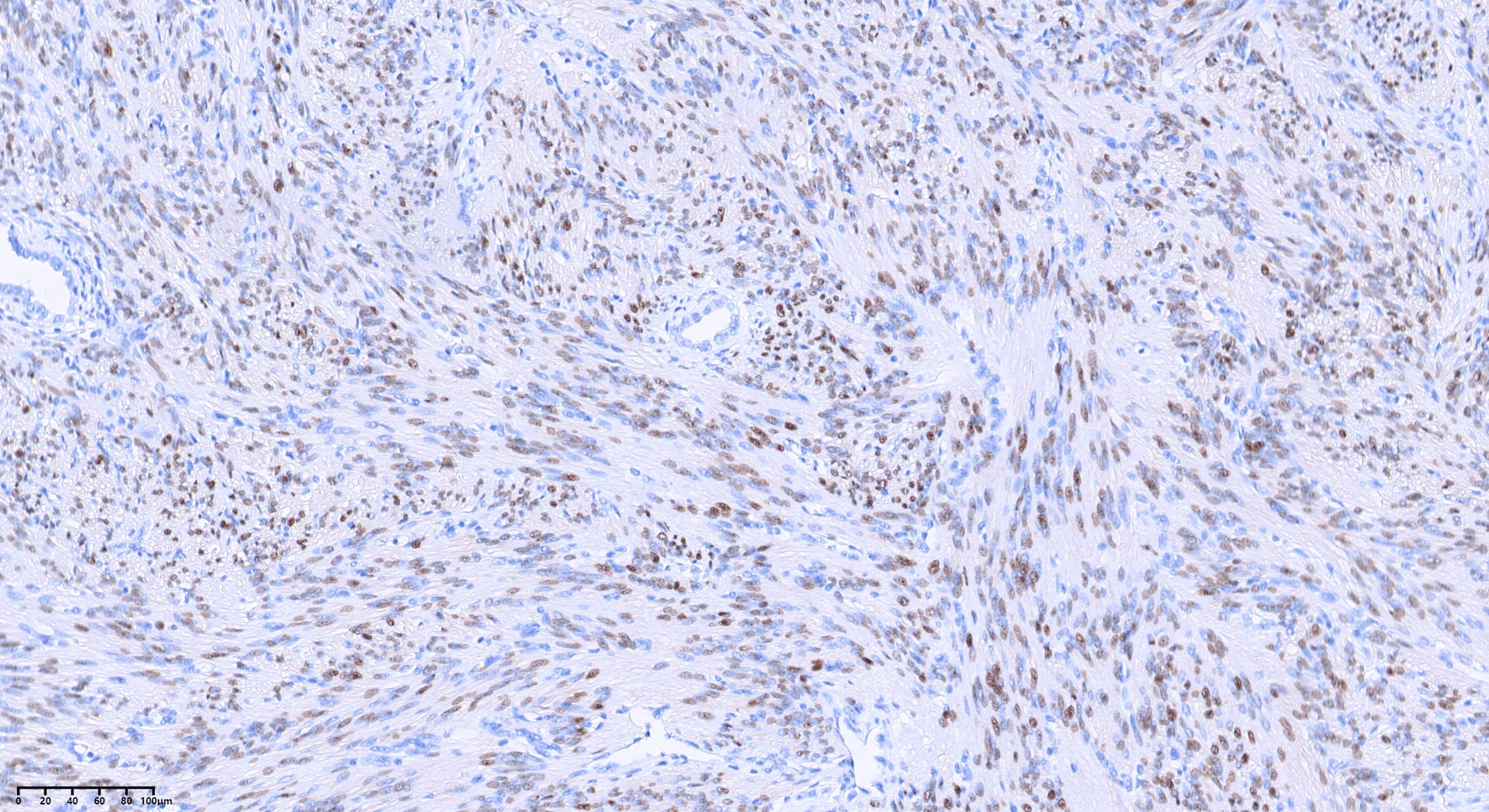


Fig.5 PR was moderately positive in the lung metastatic lesion (low power).


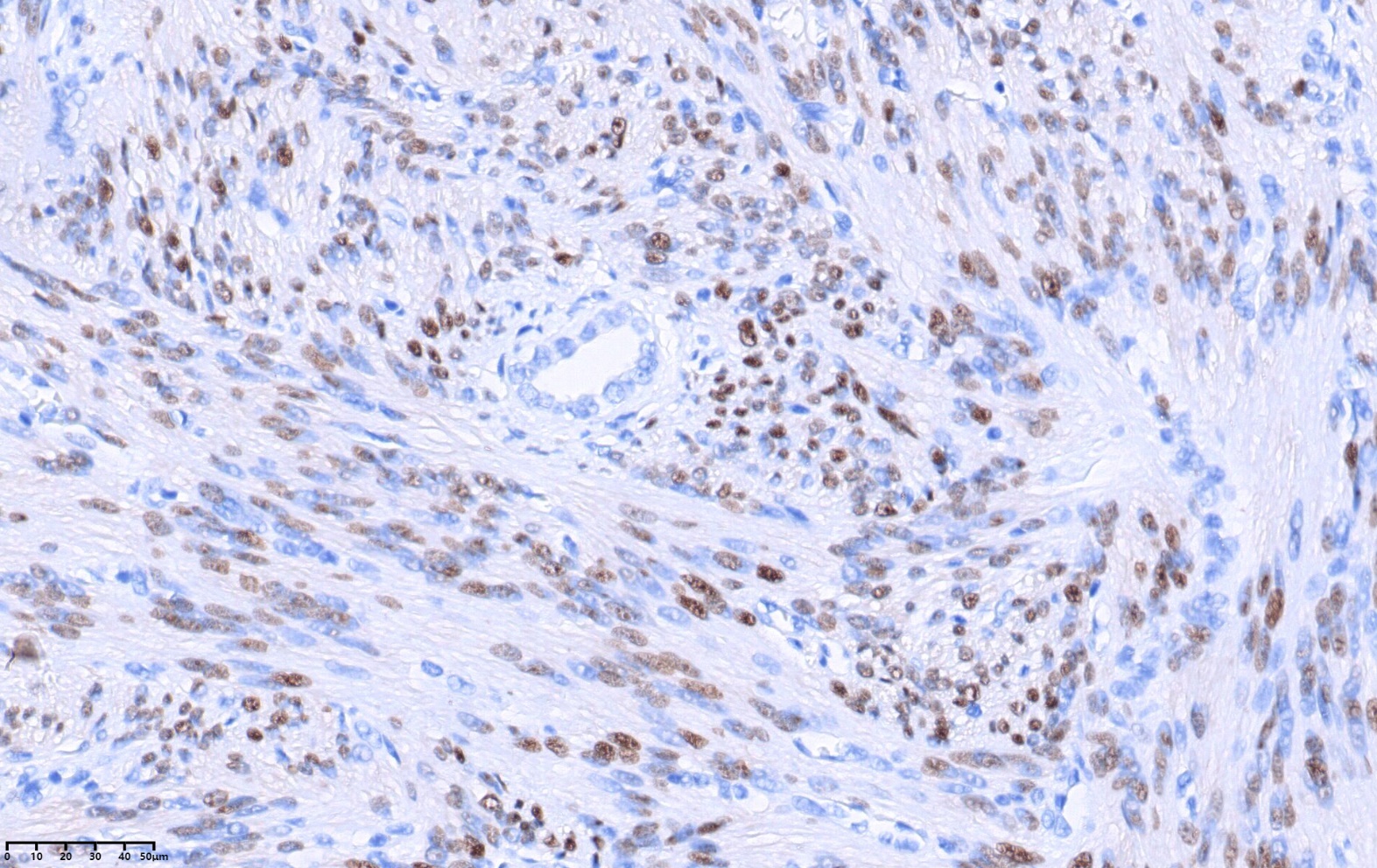


Fig.6 PR was moderately positive in the lung metastatic lesion (high power).


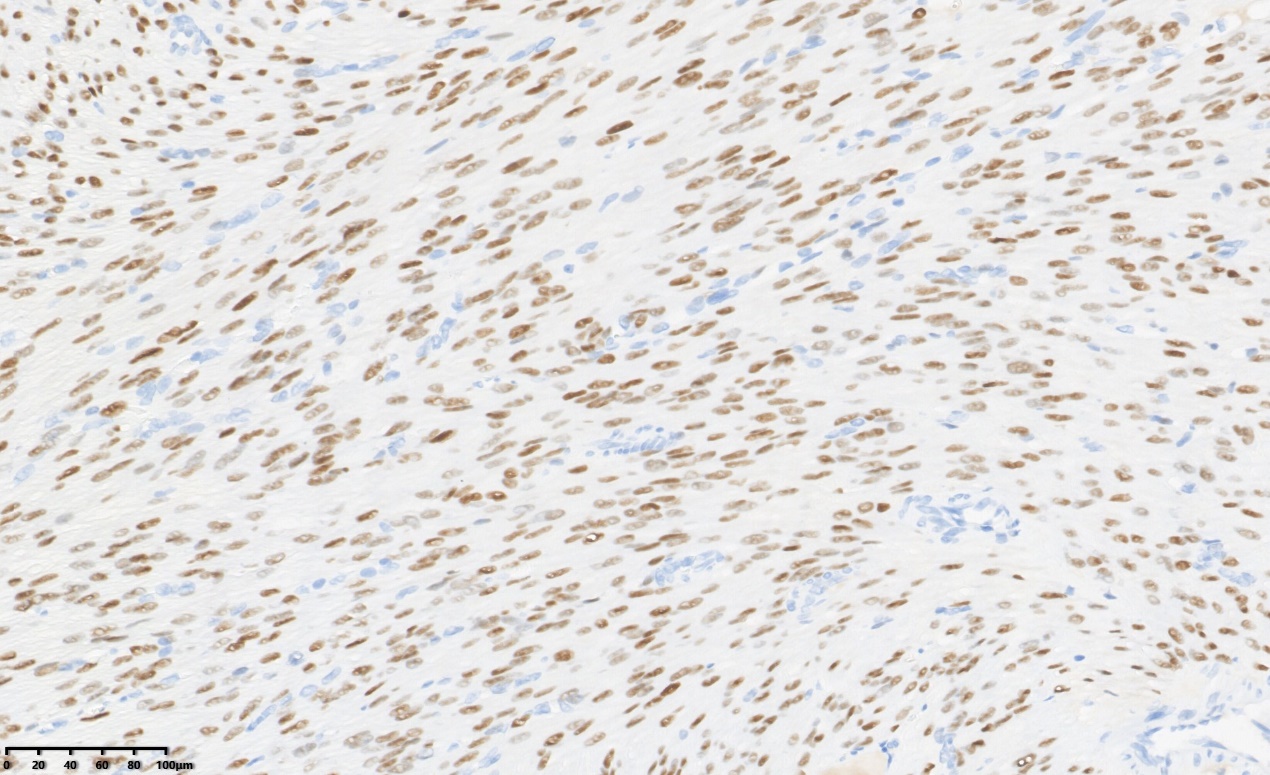


Fig.7 ER staining in the renal lesion showed diffuse strong positive expression.


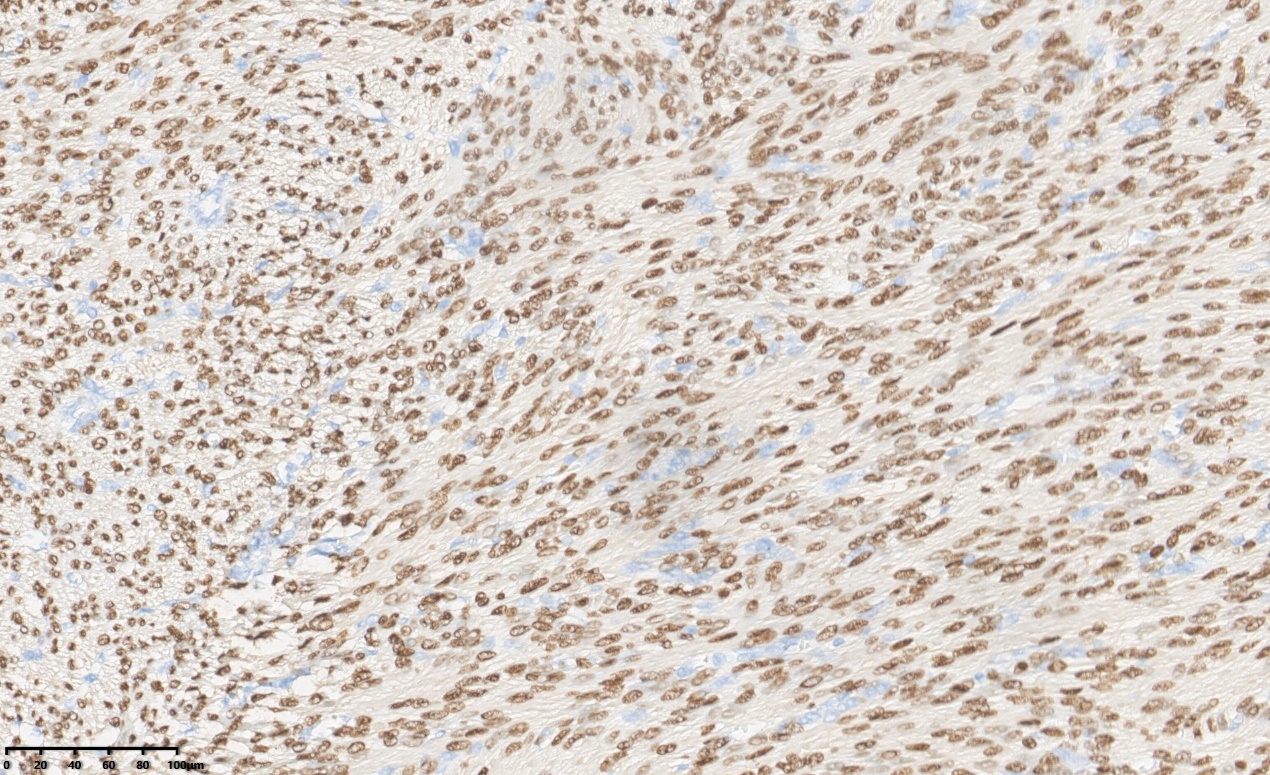


Fig.8 PR staining in the renal lesion showed diffuse strong positive expression.
